# Social Anxiety among Chinese People

**DOI:** 10.1155/2015/743147

**Published:** 2015-08-25

**Authors:** Qianqian Fan, Weining C. Chang

**Affiliations:** ^1^Division of Psychology, School of Humanities and Social Sciences, Nanyang Technological University, 14 Nanyang Drive, Singapore 637332; ^2^Department of Clinical Sciences, Graduate School of Medicine, Duke-National University of Singapore, 8 College Road, Singapore 169857

## Abstract

The experience of social anxiety has largely been investigated among Western populations; much less is known about social anxiety in other cultures. Unlike the Western culture, the Chinese emphasize interdependence and harmony with social others. In addition, it is unclear if Western constructed instruments adequately capture culturally conditioned conceptualizations and manifestations of social anxiety that might be specific to the Chinese. The present study employed a sequence of qualitative and quantitative approaches to examine the assessment of social anxiety among the Chinese people. Interviews and focus group discussions with Chinese participants revealed that some items containing the experience of social anxiety among the Chinese are not present in existing Western measures. Factor analysis was employed to examine the factor structure of the more comprehensive scale. This approach revealed an “other concerned anxiety” factor that appears to be specific to the Chinese. Subsequent analysis found that the new factor—other concerned anxiety—functioned the same as other social anxiety factors in their association with risk factors of social anxiety, such as attachment, parenting, behavioral inhibition/activation, and attitude toward group. The implications of these findings for a more culturally sensitive assessment tool of social anxiety among the Chinese were discussed.

## 1. Introduction

Social anxiety is marked by emotional discomfort, fear, apprehension, or worry about social situations, interactions with others, and being evaluated or scrutinized by other people [1]. Individuals' experience of social anxiety may vary in frequency or severity and may involve various social situations [2]. Experience of severe levels of social anxiety is accompanied by intense negative emotional reactions that would lead to avoidance and escape from interactions with significant others, acting-out, and other inappropriate coping strategies [3, 4]. Because social interaction is required in people's daily life, severe social anxiety is a debilitating condition that interferes with one's ability to enjoy a healthy social life. In addition, people with extreme social anxiety symptoms may even have higher risk for other physical or psychological comorbidities, such as substance abuse, and major depressive disorder [5, 6].

Given that social anxiety could significantly impede people's psychological wellbeing and mental health development, there is increasing empirical research on diagnosis, development, and treatment of social anxiety (e.g., [7]). Though social anxiety is recognized as a universal human condition, it might have different meanings, experiences, and manifestations in different cultures. Researchers have conducted cross-cultural studies on social anxiety to better understand specific characteristics of social anxiety in different cultures. For example, it was found that Asian Americans reported significantly more social anxiety than White Americans for both adults and adolescents [8, 9]. One study suggested that differences in prevalence rates of social anxiety across cultures might be due to differences in self-efficacy about initiating social relationships and perceived social status [10]. Another study showed that presentation and interpretation of social anxiety symptoms also vary across cultures [11].

Even though prior studies have found cross-cultural differences in social anxiety and looked into the mechanisms that might help understand these differences, some limitations of existing research need to be addressed before any firm conclusion can be drawn. First, in some of the studies [9, 12, 13], the participants were Asian Americans living in North America. Asians growing up in Western cultures as an ethnic minority in a non-Asian environment might have certain aspects different from Asian people living in Asia, where Asians are the majority. Moreover, the generic term of “Asian” could not represent the Chinese people or any single Asian ethnic group, who might have unique experiences and expressions of social anxiety. Secondly, many researchers often study Asian social anxiety from the perspective of the Westerners [14]. Therefore, their conclusions might be generated from the perspective of Westerners. For example, some cross-cultural studies directly used social anxiety scales developed in Western cultures without modifications. The results and implications might not be accurate or informative considering the fact that not only behavioral manifestations but also the linguistic expressions of social anxiety might be different across different groups. Thirdly, these studies often failed to suggest how these observed differences originated and what factors might account for them. One weakness of these studies is that they were basing their comparisons on prescribed cultural groups (demographic designation). Observed cultural differences were often interpreted as a result of distal and broad social, cultural, or contextual factors without these factors being directly examined [15]. Hence, conclusions and implications of many previous studies could only be regarded as heuristic, inspiring new hypotheses being hypothetical and tentative.

As a collective community, the Chinese emphasize interdependence with social others and the importance of maintaining social harmony [16]. Accordingly, social anxiety among the Chinese may emerge largely as a function of focused attention on how individual behaviors and performance impact others. The Chinese experience social anxiety not only in terms of the subjective experiences of the individual, but also as a concern for others. They focus their attention on how their own individual performance might impact or reflected on social others. It is important for the Chinese to take the perspective of the others in addition to that of the self to maintain a smooth coordinated existence in social situations [17]. Therefore, compared to the Westerners, the Chinese are likely to experience social anxiety with different phenomenological experiences in both the meaningful content and the magnitude. In addition, there might be specific dimensions of social anxiety showing the effect of others in the Chinese culture [18]. Hence, we would like to explore if the meanings, experiences, and manifestations of social anxiety in the Chinese might have some characteristics that are unique to the culture. We would also like to investigate whether there exist culture-specific constructs or dimensions underlying the Chinese social anxiety experiences and whether these dimensions function the same as the known factors/constructs present in the Western cultures.

In the present study, attempts have been made to address limitations of previous research to better understand social anxiety in the Chinese. First, the subjects were all Chinese. Therefore, in contrast to previous studies, where the samples were made of a mixture of Chinese and other ethnic groups, the present study was conducted on a culturally homogeneous Chinese sample in a society where the dominant culture is the Chinese culture. Secondly, all the measurements in the present study were either developed among the Chinese population or carefully reviewed and modified if necessary for those developed in the Western cultures. Therefore, unique underlying mechanisms of social anxiety conditioned in the Chinese culture are more likely to be detected with locally developed measurements [14]. Thirdly, universal and cultural specific risk factors contributing to social anxiety in Chinese population were investigated with the effect of gender controlled. Previous research has found that the lifetime prevalence rate of social anxiety disorder is 15.5% in women and 11.1% in men [19]. Females reported more severe social anxiety in more social situations than males, which is indicated by higher scores on social anxiety instruments for women [20]. Therefore, after controlling for gender, we could determine the contribution of these risk factors to social anxiety. It is helpful to understand the mechanisms of social anxiety in the Chinese culture and to further assist diagnosis, prevention, intervention, and treatment of social anxiety in the Chinese community population as well as clinical population.

Research has suggested that social anxiety is a developmental outcome of a series of risk factors, including genetic risk factors, insecure attachment experiences, inappropriate parenting styles, information processing biases, temperamental factors, and more broad contextual forces such as ethnicity and culture [21]. Previous empirical research has provided evidence on the relationships between social anxiety and these risk factors [22–24]. The current study further investigated relationships between social anxiety and four of these factors: attachment, parenting styles, BIS/BAS as the temperamental factor, and a cultural factor, patterns of interdependence. The objective was to explore the validity of the revised social anxiety scale by testing whether social anxiety could be predicted by these factors as expected.

Prior studies have indicated individuals with an avoidance attachment style tend to deny their own emotional needs for attachment and perceive others as untrustworthy, so it is difficult for them to develop intimate relationships with significant others. Individuals with anxious attachment styles may underestimate themselves and overestimate others in interpersonal relationships and further worry about abandonment and rejection [25]. Therefore, avoidance attachment would limit opportunities for intimate relationships, and anxious attachment would lead to a maladaptive pattern of thoughts and behaviors in interpersonal situations. It was hypothesized that attachment-related anxiety could predict social anxiety in a positive manner, relative to attachment-related avoidance.

Attachment theory also posits that the development of attachment styles is affected by the relationship between the individual and his caregiver. Children who have unreliable, unavailable, untrustworthy parents may have a maladaptive approach to future interpersonal relationship and cause either avoidance behaviors or demanding behaviors that may further create a chronic state of anxiety in various social situations [26]. Specifically, children are more likely to have high social anxiety if their parents are demanding and directive and value child obedience but are not responsive or supportive. Therefore, parenting characterized by low levels of warmth and high levels of control is associated with children's social anxiety. The current study hypothesized that parenting control might positively predict social anxiety, and parenting warmth might negatively predict social anxiety.

Temperamental characteristic of behavioral avoidance overlaps with shyness and social withdrawal. Previous research found that behavioral inhibition to an unfamiliar person, object, feeling, or situation is an antecedence of social anxiety [27]. Behavioral avoidance has been also shown to be associated with the development of social anxiety in people of both normal and anxious parents [28]. Therefore, the current study hypothesized that behavioral inhibition could positively predict social anxiety.

A Chinese social group is considered a vertical collective, where hierarchy is emphasized and people may sacrifice their own interests for the group [29]. Previous research has suggested that the individual is more likely to have social anxiety if he values his status, role, and interpersonal relationship in his group more than his own wellbeing [30]. Chang and Koh [31] suggested that a group may serve three functions for its individual members: meeting the functional/survival needs; meeting the affective/emotional needs; and meeting the need of having a frame of reference for defining the self. In a traditional Chinese group, people may not feel an affective interdependence to the same extent as institutional interdependence. More affective interdependence and less institutional interdependence could provide more emotional support to individuals in the group and then help them to fight with negative affect. Therefore, it was predicted that institutional interdependence and collective-self could positively predict social anxiety, and affective interdependence could negatively predict social anxiety.

Therefore, the present study aimed to investigate social anxiety among the Chinese people using a cultural psychology framework. A sequential use of qualitative and quantitative approaches was employed. The first study explored into the meaning and manifestation of social anxiety among Singaporean Chinese through interviews and focus group discussions. The second study, as a validation study, tested the relationships between risk factors and social anxiety using culturally appropriate measures. One objective was to examine whether these relationships between the newly identified dimensions converge with the existing known dimensions to form a coherent construct of social anxiety. The other objective was to test whether factors of social anxiety functioned the same in the development of social anxiety being predicted by risk factors. Finally, a measure of the cultural dimension collectivism, attitude toward group [31], was employed to examine the relationship between social anxiety and patterns of interdependence.

## 2. Method

### 2.1. Study 1

#### 2.1.1. Participants

Sixty-one unselected Chinese participants (74% male) were recruited from undergraduate courses at a large public university in Singapore in exchange for research participation credit. Among the sixty-one participants, twenty-five were interviewed individually. They ranged in age from 19 to 24 years (M = 21.00, SD = 1.98). The sample consisted of 72.0% male and 28.0% female. The remaining thirty-six participants participated in focus group discussion. They ranged in age from 20 to 33 years (M = 22.33, SD = 2.43). The sample consisted of 75.0% male and 25.0% female.

#### 2.1.2. Measures

The* Social Interaction Anxiety Scale* (SIAS; [32]) and the* Social Phobia Scale *(SPS; [32]) are both 20-item questionnaires assessing fears of social interaction or scrutiny by others. The SIAS tapped a single construct of social interaction fear. There are three factors in the SPS, including a general scrutiny concern of being observed or attracting attention in a variety of public places, specific fears, and fears of being viewed as sick, ill, odd, or having lost control in front of others.

SIAS and SPS have been translated into Chinese, and the psychometric properties have been examined in a large Chinese sample. It was suggested that the reliability coefficient was .874 for SIAS and .904 for SPS [33]. Convergent validity was also supported by a high correlation between SIAS (.514), SPS (.479), and Fear of Negative Evaluation Scale (FNE; [34]). Therefore, it was recommended that SIAS and SPS have good psychometric quality in Chinese population and could be employed to understand Chinese social anxiety.

#### 2.1.3. Procedure

Study 1 and Study 2 procedures were reviewed and approved by the Institutional Review Board (IRB) at Nanyang Technological University, Singapore (IRB number: 15-1-10-1). After signing consent forms, twenty-five participants were interviewed for half an hour. Participants were asked to speak freely and to express and clarify their experiences when they felt anxious, nervous, or awkward in social situations, such as giving a public speech and talking to strangers. If they did have such anxious experiences, they were then asked to describe their behaviors, reactions, feelings, emotions, thoughts, and anything related to their experiences. In order to gather enough information, the questions were mostly open-ended questions such as “Can you tell me more”; “Can you explain that”; “What you mean”. During this interview, participants' answers were recorded. After interviewing all the 25 people, an item pool was generated for the local expressions of social anxiety. The item pool was then compared with the items in the SIAS and the SPS. 10 new items of people's behaviors, feelings, and thoughts when they were anxious which are not in the SIAS and the SPS were generated.

The second step was to conduct focus group discussions to evaluate the new items. Seven groups were organized, and each one consisted of four to seven participants. Participants were asked to read all of the items, including 40 items in the SIAS and the SPS, and the 10 new items. Afterwards, they could ask any questions about any item, for instance, if they could understand the meanings, if they considered all of the items were related to their socially anxious behaviors, feelings, or thoughts, and so forth. Their responses were recorded. Based on the discussion of these items, some modifications were made to the 10 new items.

During the interview and focus group discussion, the communication between the interviewer and interviewees was in Chinese and English. The new items were not only represented in Chinese but also translated with back-translation method. First, the original items were translated into English by two native English speakers who have the intimate knowledge and personal experiences of both the Chinese- and English-mediated cultures. Then, the English translations were back-translated into Chinese by a native Chinese speaker. Then, the original items were compared with the back-translations, and translators made corrections to the final English translations. No items were eliminated or significantly changed during the translation process.

### 2.2. Study 2

#### 2.2.1. Participants

Two hundred and ninety-six unselected Chinese participants (32% female) were recruited from undergraduate courses at a large public university in Singapore in exchange for research participation credit. Participants ranged in age from 18 to 29 years (M = 20.78, SD = 1.73).

#### 2.2.2. Measures

The SIAS and the SPS [32], each of which consists of twenty items, plus the ten new items developed in Study 1, are to assess fears of social interaction and anxious symptoms in social situations. The whole questionnaire had an alpha coefficient of .94 in the present study. Alpha coefficients were .77 for factor 1, social interaction anxiety, .83 for factor 2, other concerned anxiety, .79 for factor 3, specific anxiety, and .90 for factor 4, being observed by others. All of the alpha coefficients are within acceptable range.

The* Experiences in Close Relationships Scale-Revised* (ECR-R; [35]) is a 36-item questionnaire to assess individual differences with respect to attachment-related anxiety and attachment-related avoidance. Attachment-related anxiety refers to an excessive need for approval from others and the fear of interpersonal rejection or abandonment, whereas attachment-related avoidance refers to an excessive need for self-reliance and fear of interpersonal intimacy or dependence. The ECR-R had an alpha coefficient of .76 in the present study.

The* Singapore Chinese Parenting Scale-short form* (Children's version) (SCPS; [36]) is a 23-item scale to measure perceived parenting. The scale was developed upon Chinese parenting dimensions, strictness (such as imposing restrictions on the child), and warmth (such as hugging, kissing, and physical nurturance). The SCPS had an alpha coefficient of .84 in the present study.

The* Behavioral Inhibition System and Behavioral Approach System Scale* (BIS/BAS; [37]) is a 24-item questionnaire to assess individual differences in the sensitivity of behavioral inhibition system and behavioral approach system. The BIS measures the sensitivity to signals of punishment, nonreward, and novelty. It functions to inhibit behaviors that may lead to negative or painful outcomes [38]. The BAS is responsible for the experience of positive feelings such as hope, elation, and happiness and sensitive to positive outcomes; behaviorally, it promotes goal-directed activities toward potential rewards [38]. The present study used the short version of BIS/BAS revised in Singapore [39]. In this 14-item revised scale, BIS/BAS included original items as well as new items developed in Singapore. The revised scale had an alpha coefficient of .73 in the present study.

The* Attitude toward Group Scale*-short version (AGS; [31]) is a 15-item scale to assess three dimensions of attitude toward group. The institutional interdependence subscale measures the degree to which the individual perceived his functional relationship with the group. The affective interdependence subscale measures the degree to which the individual sees his group as a source of and a referent for his emotions. The collective-self identification subscale measures the degree to which the individual assumes the identity of the group. The AGS had an alpha coefficient of .87 for the entire scale in the present study.

#### 2.2.3. Procedure

After participants signed an informed consent, they completed the self-report measures described above.

## 3. Results

### 3.1. Study 1

10 new items which are not in the SIAS and the SPS were shown in Table 1. All the items from the SIAS and the SPS and the 10 new items were then combined and submitted to Exploratory Factor Analysis (EFA). For the whole data, the Kaiser-Meyer-Olkin measure of the sample adequacy was .93, with Bartlett's test of sphericity significant (*P* < 0.001), indicating sufficient correlations among the variables to proceed for factor analysis. Principal Components Analysis (PCA) with promax rotation was conducted to identify underlying factors in the 50-item social anxiety scale. According to the scree plot, four-factor solution was recommended. The total variance explained by the four-factor solution was 45.40%, and by the five-factor solution it was 48.18%. In addition, the four-factor solution had more items with communities smaller than .30, so the five-factor solution was preferred. Parallel analysis was also conducted and suggested a five-factor solution because the eigenvalues of the first five factors from the current data were bigger than the 95th of the distribution of eigenvalues derived from random data. Therefore, based on the results of scree plot, parallel analysis, communalities, variance explained by factors, and rotated factor loadings by promax method, a five-factor solution was preferred.

Confirmatory factor analysis was then used to evaluate whether this five-factor model adequately fit the data [40]. Analysis of Moment Structure (AMOS) [41] was applied to test this five-factor model with all the items from the SIAS and the SPS, as well as the 10 new items. The Root Mean Square Error of Approximation (RMSEA) was .06; Comparative Fit Index (CFI) was .81. These indices indicated that a five-factor solution did not adequately fit the data [42].

Among these five factors, factor 2 and factor 5 were highly correlated (*r* = .70, *P* < 0.01). The alpha coefficients for factor 2 and factor 5 were .87 and .83, respectively, and the alpha coefficient for the combination of the two factors was .91. Therefore, the two factors seemed to measure similar aspects of social anxiety and could be combined as one factor in the final four-factor solution. Following EFA, confirmatory factor analysis was conducted to evaluate whether this four-factor model adequately fit the data [40]. The Root Mean Square Error of Approximation (RMSEA) was .07; Comparative Fit Index (CFI) was .92. These indices indicated that this four-factor solution was simpler and better than a five-factor model.

In the final four-factor solution, factor 1 included 15 items from the SIAS and one new item. According to the content of the items, factor 1 was labeled as social interaction anxiety. Factor 2 included six new items, two items from the SIAS, and one item from the SPS. Factor 2 was labeled as other concerned anxiety. Factor 3 included six items from the SPS and one new item. Factor 3 was labeled as specific anxiety. Factor 4 included three items from the SIAS, 13 items from the SPS, and two new items. Factor 4 was labeled as being observed by others.

### 3.2. Study 2

#### 3.2.1. Gender Difference in Social Anxiety

Analyses of variances were conducted to examine differences between men and women (see Table 2). Women scored higher than men on the whole social anxiety scale (*F* = 5.13, *P* < 0.05), on factor 2, other concerned anxiety (*F* = 8.35, *P* < 0.01), and on factor 4, being observed by others (*F* = 10.28, *P* < 0.01). Women and men showed no significant differences on factor 1, social interaction anxiety (*F* < 1), and factor 3, specific anxiety (*F* < 1).

#### 3.2.2. Relationships between Attachment and Social Anxiety

A series of hierarchical regression analyses were conducted to examine the specific contribution of attachment to social anxiety and its four factors. Predictor variables were entered in two steps. In the first step, gender was entered as a predictor. In the second step, attachment-related anxiety and attachment-related avoidance were simultaneously entered as predictors. This analysis provided a stringent test of the incremental validity of attachment.

The results of these analyses were presented in Table 3. In the hierarchical regression predicting social anxiety, gender was entered in the first step and explained .6% of the variance and did not account for a significant portion; *F*(1, 288) = 1.75, *P* > 0.05. In the second step, two factors of attachment explained an additional 28.1% of the variance; *F*(2, 286) = 56.45, *P* < 0.001. In the second step, attachment-related anxiety emerged as a significant predictor of social anxiety (*t*(286) = 10.01, *P* < 0.001). Attachment-related avoidance was a marginally significant predictor (*t*(286) = 1.90, *P* < 0.06). In the hierarchical regression predicting factor 1 of social anxiety, social interaction anxiety, gender explained less than .1% of the variance; *F*(1, 288) < 1. In the second step, two factors of attachment explained an additional 15.0% of the variance; *F*(2, 286) = 25.29, *P* < 0.001. Both of attachment-related anxiety and attachment-related avoidance emerged as significant predictors of factor 1 (anxiety: *t*(286) = 5.44, *P* < 0.001; avoidance: *t*(286) = 3.64, *P* < 0.001). In the hierarchical regression predicting factor 2 of social anxiety, other concerned anxiety, gender explained 1.6% of the variance and significantly predicted factor 2; *F*(1, 288) = 4.68, *P* < 0.05. In the second step, two predictors of attachment explained an additional 28.8% of the variance; *F*(2, 286) = 59.08, *P* < 0.001. In the second step, only attachment-related anxiety emerged as a significant predictor of factor 2 (*t*(286) = 10.84, *P* < 0.001). In the hierarchical regression predicting factor 3 of social anxiety, specific anxiety, gender explained .6% of the variance and did not account for a significant portion; *F*(1, 288) = 1.60, *P* > 0.05. In the second step, two predictors of attachment explained an additional 24.0% of the variance; *F*(2, 286) = 45.59, *P* < 0.001. Only attachment-related anxiety emerged as a significant predictor of factor 3 (*t*(286) = 8.99, *P* < 0.001). In the hierarchical regression predicting factor 4 of social anxiety, being observed by others, gender explained 1.7% of the variance and significantly predicted factor 4; *F*(1, 288) = 5.11, *P* < 0.05. In the second step, two predictors of attachment explained an additional 22.5% of the variance; *F*(2, 286) = 42.51, *P* < 0.001. In the second step, only attachment-related anxiety emerged as a significant predictor of factor 4 (*t*(286) = 8.92, *P* < 0.001).

#### 3.2.3. Relationships between Parenting and Social Anxiety

A series of hierarchical regression analyses were conducted to examine the specific contribution of two factors of parenting to social anxiety and its four factors. Predictor variables were entered in two steps. In the first step, gender was entered as a predictor. In the second step, parenting strictness and warmth were simultaneously entered as predictors.

The results of these analyses were presented in Table 4. In the hierarchical regression predicting social anxiety, gender was entered in the first step and explained .6% of the variance and did not account for a significant portion; *F*(1, 288) < 1. In the second step, parenting strictness and warmth explained an additional 4.2% of the variance; *F*(2, 286) = 6.35, *P* < 0.01. In the second step, only parenting strictness emerged as a significant predictor of social anxiety (*t*(286) = 3.55, *P* < 0.001). In the second hierarchical regression predicting factor 1 of social anxiety, social interaction anxiety, gender explained less than .1% of the variance; *F*(1, 288) < 1. In the second step, parenting strictness and warmth explained an additional 3.8% of the variance; *F*(2, 286) = 5.66, *P* < 0.01. In this step, only parenting strictness emerged as a significant predictor of Factor 1 (*t*(286) = 3.27, *P* < 0.01). In the hierarchical regression predicting factor 2 of social anxiety, other concerned anxiety, gender explained 1.6% of the variance and significantly predicted factor 2; *F*(1, 288) = 4.68, *P* < 0.05. In the second step, parenting strictness and warmth explained an additional 1.8% of the variance, *F*(2, 286) = 2.64, *P* > 0.05, but did not account for a significant portion. In the second step, parenting strictness emerged as a significant predictor of factor 2 (*t*(286) = 2.10, *P* < 0.05). In the hierarchical regression predicting factor 3 of social anxiety, specific anxiety, gender explained .6% of the variance and did not account for a significant portion; *F*(1, 288) = 1.60, *P* > 0.05. In the second step, parenting strictness and warmth explained an additional 5.4% of the variance; *F*(2, 286) = 8.28, *P* < 0.001. Only parenting strictness emerged as a significant predictor of factor 3 (*t*(286) = 3.98, *P* < 0.001). In the hierarchical regression predicting factor 4 of social anxiety, being observed by others, gender explained 1.7% of the variance and significantly predicted factor 4; *F*(1, 288) = 5.11, *P* < 0.05. In the second step, parenting strictness and warmth explained an additional 3.0% of the variance; *F*(2, 286) = 4.46, *P* < 0.05. In the second step, parenting strictness emerged as a significant predictor of factor 4 (*t*(286) = 2.91, *P* < 0.01).

#### 3.2.4. Relationships between BIS/BAS and Social Anxiety

A series of hierarchical regression analyses were conducted to examine the specific contribution of BIS/BAS to social anxiety and its four factors. Predictor variables were entered in two steps. In the first step, gender was entered as a predictor. In the second step, BIS and BAS were simultaneously entered as predictors.

The results of these analyses were presented in Table 5. In the hierarchical regression predicting social anxiety, gender was entered in the first step and explained .5% of the variance and did not account for a significant portion; *F*(1, 225) = 1.21, *P* > 0.05. In the second step, BIS and BAS explained an additional 17.3% of the variance; *F*(2, 223) = 23.53, *P* < 0.001. In the second step, only BIS emerged as a significant predictor of social anxiety (*t*(223) = 6.85, *P* < 0.001). In the second hierarchical regression predicting factor 1 of social anxiety, social interaction anxiety, gender explained less than .1% of the variance; *F*(1, 225) < 1. In the second step, BIS and BAS explained an additional 13.2% of the variance; *F*(2, 223) = 16.91, *P* < 0.001. In this step, both BIS and BAS emerged as a significant predictor of factor 1 (BIS: *t*(223) = 5.61, *P* < 0.001; BAS: *t*(223) = −2.91, *P* < 0.01). In the hierarchical regression predicting factor 2 of social anxiety, other concerned anxiety, gender explained 1.9% of the variance and significantly predicted factor 2; *F*(1, 225) = 4.25, *P* < 0.05. In the second step, BIS and BAS explained an additional 15.3% of the variance; *F*(2, 223) = 20.55, *P* < 0.001. In the second step, only BIS emerged as a significant predictor of factor 2 (*t*(223) = 6.40, *P* < 0.001). In the hierarchical regression predicting factor 3 of social anxiety, specific anxiety, gender explained .8% of the variance and did not account for a significant portion; *F*(1, 225) = 1.91, *P* > 0.05. In the second step, BIS and BAS explained an additional 5.5% of the variance; *F*(2, 223) = 6.58, *P* < 0.01. BIS emerged as a significant predictor of factor 3 (*t*(223) = 3.53, *P* < 0.001). In the hierarchical regression predicting factor 4 of social anxiety, being observed by others, gender explained 1.6% of the variance and did not significantly predict factor 4; *F*(1, 225) = 3.55, *P* > 0.05. In the second step, BIS and BAS explained an additional 14.9% of the variance; *F*(2, 223) = 19.89, *P* < 0.001. In the second step, only BIS emerged as a significant predictor of factor 4 (*t*(223) = 6.25, *P* < 0.001).

#### 3.2.5. Relationships between Attitude toward Group and Social Anxiety

A series of hierarchical regression analyses were conducted to examine the specific contribution of attitude toward group to social anxiety and its four factors. Predictor variables were entered in two steps. In the first step, gender was entered as a predictor. In the second step, three factors of attitude toward group were simultaneously entered as predictors.

The results of these analyses were presented in Table 6. In the hierarchical regression predicting social anxiety, gender was entered in the first step and explained .6% of the variance and did not account for a significant portion; *F*(1, 288) = 1.75, *P* > 0.05. In the second step, three factors of attitude toward group explained an additional 3.7% of the variance; *F*(3, 285) = 3.62, *P* < 0.05. In the second step, affective interdependence and collective-self identification emerged as significant predictors of social anxiety (affective interdependence: *t*(285) = −2.62, *P* < 0.01; collective-self identification: *t*(285) = 2.42, *P* < 0.05). In the second hierarchical regression predicting factor 1 of social anxiety, social interaction anxiety, gender explained less than .1% of the variance; *F*(1, 288) < 1. In the second step, three factors of attitude toward group explained an additional 3.6% of the variance; *F*(3, 285) = 3.59, *P* < 0.05. In this step, only affective interdependence emerged as a significant predictor of factor 1 (*t*(285) = −3.10, *P* < 0.01). In the hierarchical regression predicting factor 2 of social anxiety, other concerned anxiety, gender explained 1.6% of the variance and significantly predicted factor 2; *F*(1, 288) = 4.68, *P* < 0.05. In the second step, three factors of attitude toward group explained an additional 2.7% of the variance; *F*(3, 285) = 2.69, *P* < 0.05. In the second step, collective-self identification emerged as a significant predictor of factor 2 (*t*(285) = 2.13, *P* < 0.05). In the hierarchical regression predicting factor 3 of social anxiety, specific anxiety, gender explained .6% of the variance and did not account for a significant portion; *F*(1, 288) = 1.60, *P* > 0.05. In the second step, three factors of attitude toward group explained an additional 7.2% of the variance; *F*(3, 285) = 7.46, *P* < 0.001. All of the three factors of attitude toward group emerged as significant predictors of factor 3 (institutional interdependent: *t*(285) = 2.39, *P* < 0.05; affective interdependence:*t*(285) = −4.14, *P* < 0.001; collective-self identification: *t*(285) = 2.42, *P* < 0.05). In the hierarchical regression predicting factor 4 of social anxiety, being observed by others, gender explained 1.7% of the variance and significantly predicted factor 4; *F*(1, 288) = 5.11, *P* < 0.05. In the second step, three factors of attitude toward group explained an additional 2.2% of the variance; *F*(3, 285) = 2.22, *P* > 0.05. In the second step, only collective-self identification emerged as a significant predictor of factor 4 (*t*(285) = 2.11, *P* < 0.05).

## 4. Discussion

The SIAS and the SPS were developed in the Western context. The ten new items developed from qualitative interviews from Chinese participants were not present in the original Western constructed scales, and they represent culture-specific social anxious symptoms of the Chinese people. The revised social anxiety scales consisted of 50 items, including 40 items from the SIAS and the SPS and ten new items generated from the interview and focus group discussions among the Chinese people in a predominantly Chinese society, Singapore.

Among the 10 new items in the present study, two items are physical symptoms people experience with others around. One is “I feel that I need to take a deep breath when I am with others”; the other is “I stutter when I make a public speech.” Two items are symptoms of being afraid of making others uncomfortable: one is “I am afraid of making others uncomfortable if I do not know them well”; the other is “I am afraid my inappropriate behaviors may cause stress in my friends.” The remaining six items are symptoms if people could maintain their (or their family's) good image consistently in front of others: (1) I am afraid of being singled out to deal with difficulties; (2) I am worried people will laugh at my anxious behaviors; (3) I am afraid of being seen as a person with no proper upbringing; (4) I am worried that I could not always maintain a good image; (5) I am afraid of losing my friends if I behave in a wrong way; (6) I am afraid of being misunderstood as a loner.

According to the results of EFA and CFA, a four-factor structure best represents the constructs of the 50-item combined social anxiety scale. Factor 1 is social interaction anxiety measuring anxious symptoms while interacting with others. Factor 2 is other concerned anxiety, and most of the items in this factor were generated in the present study. Factor 2 assesses whether people's performance would make others uncomfortable or influence others in an unbeneficial way. Factor 3 is specific anxiety measuring specific anxiety in certain situations, like using public toilets, drinking with a group of people, and sitting facing people on a bus or a train. Factor 4 was labeled being observed by others assessing whether an individual or his anxious behaviors could be noticed by others. Previous factor analysis of social anxiety has found factors 1, 3, and 4, but factor 2, other concerned anxiety, was only extracted in the current study [43, 44]. The Chinese culture promotes the interdependent self, so individuals are reminded to be sensitive to the needs and expectations of the group, the others, rather than the individual self [45]. Traditionally, the Chinese culture emphasizes obligations to family (filial piety), making people worried whether their inappropriate behaviors may exert bad influences to or reflect badly on their family or their friends [46–48].

Study 2 examined the relationship between social anxiety and its risk factors, attachment, parenting, BIS, and patterns of interdependence. The results of Study 2 have supported the validity of the revised social anxiety scale and provided further evidence to understand the mechanisms of social anxiety from a cultural perspective. It was suggested that the Chinese culture-specific factor, other concerned anxiety, plays an important role as the other factors in the original social anxiety scale in being predicted by theoretically risk factors.

The results of predicting the role of attachment in social anxiety suggested that the association between social anxiety and attachment was independent of gender difference in social anxiety. Hierarchical regression analyses showed that attachment-related anxiety remained a more significant predictor of social anxiety and its four factors relative to attachment-related avoidance. The results implicated that social anxiety might be more likely experienced when people have an excessive need for approval from others and fear of interpersonal rejection or abandonment. However, the excessive need for self-reliance and fear of interpersonal intimacy or dependence may not operate as a relative vulnerability factor for the development of social anxiety. Therefore, attachment-related anxiety appears to better predict social anxiety symptoms.

In addition, hierarchical regression analyses showed that parenting strictness predicted social anxiety and its four factors. The finding was consistent with prior research that anxious symptoms were positively associated with rejecting and controlling parenting [49]. Furthermore, Chinese parenting has its own cultural specific characteristics as well as the general features found in Western cultures. In the Chinese parenting meaning system, parents are expected to be strict to their children in regulating the child's behavior and place a high emphasis and value on child's obedience to parental wishes [47]. Parents control their children's behaviors through the strict use of rules and regulations initiated by the parents. The strictness, however, is usually meant for the “good” of the child, to assist children's self-development and success [36]. Nevertheless, the present study found that strictness, though including some cultural specific manifestations, could also lead to more experiences of social anxiety, which may further cause more mental health problems in the long run. In addition, warmth, showing parents' love and support to children, was not significantly correlated with social anxiety. This finding was not consistent with prior research. Some previous research suggested that social anxiety in the child was associated with a parenting style characterized by high levels of low warmth [50]. Therefore, low warmth is supposed to increase social anxiety, whereas high warmth may function as a protective factor to reduce social anxious symptoms. The inconsistency may be due to the two intertwined concepts strictness and warmth in Chinese parenting meaning system. Both strictness and warmth are manifested in activities initiated by parents with the parent as the locus of decision. Parents are expected to take care of children of all ages to the extent of sacrificing their own needs. However, such care-taking is based on decisions made by the parents to ensure that only the best is provided for the children [51]. Furthermore, there is no clear distinction between appropriate warmth and overprotection or overinvolvement, which may also contribute to social anxiety [50].

Then, the relationship between BIS/BAS and social anxiety was also examined in the present study. Hierarchical regression analyses showed that only BIS emerged as a stronger predictor of social anxiety and its four factors than BAS. This finding was consistent with prior research on relationships between BIS and anxiety that behavioral inhibition is associated specifically with the development of social anxiety [52]. The present results suggested that individuals are more likely to experience social anxiety if they are more sensitive to signals of punishment, nonreward, and novelty in interacting with social others.

With the relationships between risk factors above and social anxiety being examined, we would like to explore further whether social anxiety could be predicted by a cultural factor, patterns of interdependence. Patterns of interdependence were measured by attitude toward group scale. Hierarchical regression analyses showed that three factors of attitude toward group, institutional interdependence, affective interdependence, and collective-interdependence, significantly predicted social anxiety and its four factors. Among the three factors of attitude toward group, collective-self contributed more than affective interdependence, which contributed more than institutional interdependence. The positive relationship between institutional interdependence and social anxiety suggested that the more an individual perceived his functional relationship with the group, the more he would experience anxiety. The negative relationship between affective interdependence and social anxiety suggested that the more the people see their group as a source of a referent for their emotions, the more they are less likely to feel anxious. The third factor, collective-self, could predict social anxiety in a positive manner, indicating that the more one assumes the identity of the group, the more one's self-identity might be temporarily suspended but lead to more anxious symptoms. Therefore, in Chinese culture, when an individual's interests are in conflict with those of the group, the individual, as a moral principle, is expected to sacrifice his personal interests. However, restraining personal interests and feelings would lead to more negative affect, for example, anxiety.

## 5. Implications and Limitations

The present study suggested that social anxiety has unique meanings and manifestations in the Chinese culture, which is indicated by the new factor identified in the revised social anxiety scales. Similar to the other three factors, the new factor is a necessary component of social anxiety among Chinese people. Therefore, social anxiety could be more comprehensively measured with cultural specific manifestations, in addition to universal manifestations. The current findings also suggested that attachment, parenting styles, BIS/BAS, and patterns of interdependence all served as risk factors of social anxiety. People with an excessive need for approval from others and fear of interpersonal rejection or abandonment would be more likely to have social anxiety. Parenting strictness predicted social anxiety positively; however, low level of parenting warmth did not prevent the development of social anxiety in the Chinese culture. As in the Western culture, behavioral inhibition also predicted social anxiety positively. Furthermore, from the perspective of interdependence, the present study suggested that Chinese people may tend to sacrifice their own interests for the interests of collective. However, suppression of individual's own needs might result in high social anxiety.

Although the current study highlights the importance of the cultural specific factor of social anxiety among Chinese people, limitations of the study should be considered when interpreting the findings. First, the participants consisted of a small sample of undergraduate students without significant social anxiety. This convenient sample made it difficult to generalize the findings to a general population or those with significant difficulties in interacting with people. Second, the culture of Singapore is a mix of Chinese, Malay, Indian, and British cultures. Singaporean culture is different from Western cultures but also different from traditional Chinese culture. Cautions should be exercised when applying the current findings to other Chinese populations in different societies. In addition, the sample size to item ratio (5 : 1) was small to conduct an Exploratory Factor Analysis. The current 4-factor solution might not capture factor structure of the revised social anxiety scale correctly. Therefore, future research employing a larger sample size may further current understanding of social anxiety manifestations among Chinese people. Moreover, the present study is a cross-sectional study of social anxiety. To capture the development of social anxiety and its risk factors, such as attachment and parenting, a longitudinal study would be more reliable to detect the development of each construct and the relationships between these constructs.

## Figures and Tables

**Table 1:** 10 new items of social anxiety scale.

1	I am afraid of being singled out to deal with difficulties.
2	I am worried people will laugh at my anxious behaviors.
3	I am afraid of making others uncomfortable if I do not know them well.
4	I am afraid of being seen as a person with no proper upbringing.
5	I am worried that I could not always maintain a good image.
6	I am afraid of losing my friends if I behave in a wrong way.
7	I feel that I need to take a deep breath when I am with others.
8	I am afraid my inappropriate behaviors may cause stress in my friends.
9	I stutter when I make a public speech.
10	I am afraid of being misunderstood as a loner.

**Table 2:** Mean difference between women and men on social anxiety and its 4 factors.

	Men			Women			*F*	*P*
	*n*	M	SD	*n*	M	SD		
Social anxiety	128	3.36	.91	228	3.57	.78	5.13	0.02^*^
F1	128	3.59	1.09	228	3.68	.85	.77	0.38
F2	128	3.59	1.10	228	3.92	.99	8.35	0.00^**^
F3	128	2.49	.87	228	2.44	.90	.17	0.68
F4	128	3.39	1.04	228	3.73	.94	10.28	0.00^**^

Note: ^*^ *P* < 0.05, ^**^ *P* < 0.01. F1 = social interaction anxiety; F2 = other concerned anxiety; F3 = specific anxiety; F4 = being observed by others.

**Table 3:** Specificity of factors of attachment in predicting social anxiety and its four factors.

Measures	*R* ^2^	*B*	SE *B*	*β*	*t*
Predicting social anxiety	.281^***^				
Attachment-related anxiety		.414	.041	.506	10.011^***^
Attachment-related avoidance		.088	.046	.097	1.903^‡^
Predicting social interaction anxiety (F1)	.150^***^				
Attachment-related anxiety		.279	.051	.301	5.444^***^
Attachment-related avoidance		.209	.057	.202	3.640^***^
Predicting other concerned anxiety (F2)	.288^***^				
Attachment-related anxiety		.565	.052	.542	10.836^***^
Attachment-related avoidance		−.052	.059	−.045	−.888
Predicting specific anxiety (F3)	.240^***^.				
Attachment-related anxiety		.426	.047	.468	8.991^***^
Attachment-related avoidance		.092	.053	.090	1.725
Predicting being observed by others (F4)	.225^***^				
Attachment-related anxiety		.454	.051	.465	8.920^***^
Attachment-related avoidance		.050	.057	.046	.871

Note: ^‡^ *P* < 0.06, ^***^ *P* < 0.001.

**Table 4:** Specificity of factors of parenting in predicting social anxiety and its four factors.

Measures	*R* ^2^	*B*	SE *B*	*β*	*t*
Predicting social anxiety	.042^**^				
Parenting strictness		.223	.063	.207	3.547^***^
Parenting warmth		−.010	.060	−.010	−.174
Predicting social interaction anxiety (F1)	.038^**^				
Parenting strictness		.234	.072	.192	3.267^**^
Parenting warmth		−.086	.068	−.074	−1.261
Predicting other concerned anxiety (F2)	.018				
Parenting strictness		.170	.081	.123	2.097^*^
Parenting warmth		.049	.077	.037	.630
Predicting specific anxiety (F3)	.054^***^				
Parenting strictness		.277	.070	.231	3.979^***^
Parenting warmth		.019	.066	.016	.281
Predicting being observed by others (F4)	.030^*^				
Parenting strictness		.219	.075	.170	2.915^**^
Parenting warmth		.016	.072	.013	.223

Note: ^*^ *P* < 0.05, ^**^ *P* < 0.01, and ^***^ *P* < 0.001.

**Table 5:** Specificity of factors of BIS/BAS in predicting social anxiety and its four factors.

Measures	*R* ^2^	*B*	SE *B*	*β*	*t*
Predicting social anxiety	.173^***^				
BIS		.510	.074	.438	6.854^***^
BAS		−.119	.065	−.116	−1.837
Predicting social interaction anxiety (F1)	.132^***^				
BIS		.485	.086	.369	5.612^***^
BAS		−.218	.075	−.189	−2.912^**^
Predicting other concerned anxiety (F2)	.153^***^				
BIS		.612	.096	.411	6.405^***^
BAS		−.121	.083	−.093	−1.465
Predicting specific anxiety (F3)	.055^**^				
BIS		.315	.089	.241	3.526^***^
BAS		−.011	.077	−.009	−.138
Predicting being observed by others (F4)	.149^***^				
BIS		.558	.089	.403	6.254^***^
BAS		−.071	.077	−.058	−.914

Note: ^**^ *P* < 0.01, and ^***^ *P* < 0.001.

**Table 6:** Specificity of factors of attitude toward group in predicting social anxiety and its four factors.

Measures	*R* ^2^	*B*	SE *B*	*β*	*t*
Predicting social anxiety	.037^*^				
Institutional interdependence		.067	.080	.071	.837
Affective interdependence		−.192	.073	−.211	−2.621^**^
Collective-self identification		.160	.066	.194	2.418^*^
Predicting social interaction anxiety (F1)	.036^*^				
Institutional interdependence		.036	.091	.034	.400
Affective interdependence		−.258	.083	−.250	−3.099^**^
Collective-self identification		.135	.075	.143	1.785
Predicting other concerned anxiety (F2)	.027^*^				
Institutional interdependence		.067	.102	.055	.654
Affective interdependence		−.112	.094	−.096	−1.197
Collective-self identification		.180	.085	.171	2.130^*^
Predicting specific anxiety (F3)	.072^***^				
Institutional interdependence		.209	.088	.198	2.387^*^
Affective interdependence		−.332	.080	−.327	−4.144^***^
Collective-self identification		.175	.072	.190	2.416^*^
Predicting being observed by others (F4)	.022				
Institutional interdependence		.039	.096	.035	.410
Affective interdependence		−.119	.088	−.109	−1.360
Collective-self identification		.168	.079	.170	2.115^*^

Note: ^*^ *P* < 0.05, ^**^ *P* < 0.01, and ^***^ *P* < 0.001.
